# Properdin has an ascendancy over factor H regulation in complement-mediated renal tubular damage

**DOI:** 10.1186/1471-2369-15-82

**Published:** 2014-05-22

**Authors:** Seiji Nagamachi, Isao Ohsawa, Hiyori Suzuki, Nobuyuki Sato, Hiroyuki Inoshita, Atsuko Hisada, Daisuke Honda, Mamiko Shimamoto, Yoshio Shimizu, Satoshi Horikoshi, Yasuhiko Tomino

**Affiliations:** 1 Division of Nephrology, Department of Internadl Medicine, Juntendo University Faculty of Medicine, Tokyo, Japan

## Abstract

**Background:**

Urinary (U)-complement components have been detected in patients with proteinuric renal diseases, and complement activation via the alternative pathway (AP) is believed to play a role in renal tubular damage. The present study aimed to examine the regulation of complement AP activation in patients with renal tubular damage by focusing on the balance between properdin (P) and factor H (fH).

**Methods:**

In the *in vivo* studies, U concentrations of P, fH and membrane attack complex (MAC) were measured in patients with renal diseases using an enzyme-linked immunosorbent assay (ELISA), and their relationships with the clinical data were evaluated. In the *in vitro* studies, human proximal tubular epithelial cells (PTECs) were incubated with normal human serum (NHS), P-depleted serum (PDS), purified P and/or fH. Changes in cell morphology and phenotype were assessed by microscopy, real-time polymerase chain reaction (PCR), immunostaining and a cell viability assay.

**Results:**

The U-P, fH and MAC concentrations were significantly higher in patients with renal disease than in normal controls and correlated with the U-protein and tubular damage markers. Furthermore, multivariate analysis revealed a relationship between P levels and tubular damage markers. There were no significant changes in morphology and mRNA expression in the AP components (P, fH, fB, C3, C5 and C9) after the addition of up to 25% NHS. Dose-dependent depositions of P or fH were observed after the addition of P or fH on PTECs. Depositions of P were not inhibited by fH in a mixture of a fixed concentration of P and a variable concentration of fH, and vice versa. Preincubation with the fixed concentration of P before the addition of NHS or PDS increased the depositions of P, C3 and MAC compared with incubation with intact NHS or intact PDS only; the depositions of C3 and MAC showed a serum-dependent trend. Preincubation with P before NHS addition significantly suppressed cell viability without causing morphological changes.

**Conclusions:**

In the pathogenesis of renal tubular damage, P can directly bind to PTECs and may accelerate AP activation by surpassing fH regulation.

## Background

Both experimental and clinical studies have shown that proteinuria can directly lead to tubulointerstitial injury [1,2]. Urinary (U)-complement components have been detected in patients with proteinuric renal diseases [3-8], and complement activation via the alternative pathway (AP) may be involved in renal tubular damage [9-12].

The AP is one of three complement activation pathways: the classical pathway (CP), the lectin pathway (LP) and the AP. The CP and LP are initiated by specific molecules, namely antibodies and carbohydrates, respectively. The AP remains continuously activated at low levels (the so-called “tick over” effect) and acts indiscriminately on any type of surface, i.e. native cells, tissues and foreign cells or particles [13]. Therefore, the AP can be controlled by a number of inhibitory regulators; however, there is only one known positive regulator called properdin (P). AP activation is amplified after the formation of the C3 convertase complex (C3bBb). P can bind to this complex and stabilize it by 5–10 fold [14]. Factor H (fH), which is one of the most important fluid phase inhibitory regulators of AP, serves as a cofactor for factor I in the facilitation of the cleavage of C3b to inactive C3b; it also accelerates the decay of C3b, Bb and C3bBbP [13]. Inadequately controlled AP activation has been implicated in the pathogenesis of different diseases. In addition, researchers have recognized that mutated or defective fH may be associated with atypical haemolytic uremic syndrome, dense deposit disease and age-related macular degeneration [15].

Recent studies have shed new light on P, which can also act as an initiator of direct complement activation by recognizing and binding to specific target surfaces such as apoptotic T cells [16] and proximal tubular epithelial cells (PTECs) [9]; this mechanism has been referred to as the properdin-directed pathway (PDP) [17]. On the other hand, fH can also act as a surface-bound regulator of AP and can bind to endothelial cells [18] and PTECs [19]. P and fH are key counterpart regulator proteins for AP, and interestingly, recent reports have indicated that both can bind to renal PTECs [10,14,19].

Recent reports have also suggested that both P and fH are involved in the pathogenesis of complement-mediated renal tubular damage. However, most of the previous reports were either research studies that evaluated U-complement components in patients with a single renal disease such as IgA nephropathy [3], membranous nephropathy [4] and others or studies that evaluated either U-P or U-fH in patients with proteinuric renal diseases without examining the combination of P and fH in patients with a variety of renal diseases. In addition, superiority between P and fH in the AP activation of PTECs remains unclear; the competitive binding of P and fH on PTECs has not been fully studied.

In the present study, we planned *in vivo* and *vitro* studies to examine the abovementioned issues. In the *in vivo* studies, we measured the U-complement components (P, fH and MAC) in patients with various renal diseases and examined their relationships with the clinical data. In the *in vitro* studies, we used cultured human PTECs to examine AP activation during renal tubular damage by focusing on the competitive binding of P and fH and the enhancement effects of P.

## Methods

### *In vivo* studies

#### Patients and controls

Sixty-three patients with renal diseases (35 males and 28 females; mean age, 42.3 ± 17.2 years) who were referred to the Juntendo University Hospital between July 2010 and November 2012 and 48 healthy volunteers (normal controls; 34 males and 14 females; mean age, 34.1 ± 5.5 years) with no history of renal dysfunction in annual periodic medical check-ups were enrolled. Renal diseases included the following: IgA nephropathy (IgAN; n = 25; males/females, 13/12; age, 38.0 ± 12.4 years), membranous nephropathy (MN; n = 6, 3/3, 60.1 ± 13.7 years), non-IgA mesangial proliferative glomerulonephritis (Non-IgAN; n = 5, 5/0, 44.4 ± 27.7 years), minimal change nephrotic syndrome (MCNS; n = 5, 4/1, 46.2 ± 28.3 years), membranoproliferative glomerulonephritis (MPGN; n = 2, 1/1, 48.0 ± 21.2 years), focal segmental glomerulosclerosis (FSGS; n = 2, 2/0, 32.5 ± 21.9 years), tubulointerstitial nephritis (n = 1, 0/1, 33.0 years), lupus nephritis (LN; n = 8, 5/3, 31.3 ± 6.9 years), diabetes mellitus-induced nephropathy (DMN; n = 3, 3/0, 46.6 ± 25.1 years), nephrosclerosis (n = 2, 2/0, 57.0 ± 1.4 years), Alport syndrome (n = 2, 0/2, 44.5 ± 2.1 years), light chain deposition disease (n = 1, 1/0, 52.0 years) and amyloidosis (n = 1, 1/0, 65.0 years). This study was approved by the Institutional Human Study ethics committee at Juntendo University, and written informed consent was obtained from all participants. Each histological diagnosis was classified through routine examination, including light microscopy (LM) of renal biopsy specimens, and through the results of immunoglobulin and complement deposition as assessed by immunofluorescence (IF) and electron microscopy.

### Laboratory data

Serum creatinine (S-Cre), U-creatinine (U-Cre), estimated glomerular filtration rate (eGFR), U-protein, U-beta-2-microglobulin (U-B2MG) and U-N-acetyl-beta-D-glucosaminidase (U-NAG) levels were measured as part of routine clinical analysis at the time of renal biopsy. Laboratory data were collected in the central laboratory at the Juntendo University Hospital.

### Measurement of P, fH and MAC in the urine

Urine samples were obtained and stored at −80°C before use. U-concentrations of P, fH and MAC were measured with commercially available ELISA kits [Human Properdin KIT and Human Factor H KIT, Hycult Biotech, Uden, Netherlands and SC5b-9 (MAC) Plus EIA KIT, Quidel, San Diego, CA, USA]. Absorbance at 450 nm was determined using a microplate reader (SpectraMax 340, Molecular Devices, Sunnyvale, CA, USA).

### *In vitro* studies

#### Blood samples and reagents

Normal human serum (NHS) was obtained from a healthy volunteer. P-depleted human serum (PDS) and purified P were purchased from CompTech (Tyler, Texas, USA), while purified fH was purchased from Calbiochem (Billerica, MA, USA). This study was also approved by the Institutional Human Study ethics committee at Juntendo University, and written informed consent was obtained from this participant.

### Cell culture and preparation

The immortalized renal PTEC line human kidney-2 (HK-2) was purchased from ATCC (Manassas, VA, USA). PTECs were cultured until they were 90%–95% confluent on the cover glass in 12-well culture plates for the IF studies and microscopic analysis, in a 100-mm dish for RNA isolation and cDNA synthesis or in 96-well culture plates for cell viability studies using a medium (DMEM; SIGMA, St. Louis, MA, USA) containing 10% fetal calf serum. The cells on the culture plates were incubated with an NHS-containing medium, P-depleted serum (PDS), P and/or fH for 3 h after incubation with a serum-free medium in the CO_2_ incubator (5% CO_2_) at 37°C for 24 h, as described previously [9].

### Microscopic analysis

To determine the adequate conditions for the study and assess the influence of complement activation and the enhancement effects of P, PTECs were incubated with different concentrations of NHS (5% and 25%) for 3 h with or without preincubation with P (5 μg/mL) for 3 h. The morphological changes in PTECs were observed by LM (Keyence All-in-one fluorescence microscope BZ-9000, Keyence, Osaka, Japan).

### mRNA measurement of complement components belonging to the AP

To further confirm adequate conditions for this study, we examined the mRNA expression of the complement components that belonged to the AP of PTECs. PTECs were incubated with different concentrations of NHS (5% and 25%) for 3 h. The RNA of PTECs was isolated using an RNeasy Minikit (Qiagen, Valencia, CA, USA) according to the manufacturer’s instructions. For cDNA synthesis, 4 μL of MTP, 2 μL of random primer and 2 μg of RNA were incubated at 70°C for 3 min and directly cooled. cDNA was synthesized by adding a mixture containing 0.5 μL of M-MULVRT, 1 μL of RNase inhibitor and 2 μL of 10× real-time polymerase chain reaction (RT-PCR) buffer. The mixture was incubated at 42°C for 1 h. The samples were stored at −20°C. RT-PCR was performed in a reaction of 18 μL containing 10 μL of SYBR Green mastermix (Applied Biosystems, Foster City, CA, USA), 1.6 μL of each primer, 4.8 μL of nuclease-free water and 10 ng of cDNA. All samples were analysed in triplicate. Thermal cycling was performed on an Applied Biosystems 7500 Fast RT-PCR system (Applied Biosystems), with a hot start at 95°C for 20 s. Subsequently, 40 cycles were performed, which included a denaturation step at 95°C for 3 s followed by an annealing step at 60°C for 30 s. A last step was included to detect the formation of primer dimers (melting curve), starting with 15 s at 95°C followed by 60 s at 60°C, 15 s at 95°C and 15 s at 60°C for one cycle. cDNA-free double-distilled water was included as a negative control in each reaction. For quantification of the PCR product, the samples were standardised with the PCR product for glyceraldehyde-3-phosphate dehydrogenase (GAPDH). The primers were designed with Primer Express software (Life Technologies, Carlsback, CA, USA). The following primers were used: human P, forward 5′-GCATCCAGCACTGCCCCTTGAAA-3′ and reverse 5′-GGCACGGGTAGGATTAGGTCCACA-3′; human fH, forward 5′-TCCAGAAGGCACCCAGGCTATCTA-3′ and reverse 5′-CCACAGGGCCTTTTCTGACATTTCC-3′; human factor B, forward 5′-CCATTTGTCTCCCCTGCACCGA-3′ and 5′-GCAGCTCTTCCTTTTGTTGCTGGC-3′; human C3, forward 5′-ACGCGCAAGGGGATGTTCCA-3′ and reverse 5′-TCCCTGTTGGCTGGGATCGTGA-3′; human C5, forward 5′-CTTGGGAGGCCAGTAGAGGTGCT-3′ and reverse 5′-TGTACTGTAGCCAAGCCACTGCCAA-3′; human C9, forward 5′-ACACGACCAGTTATGACCCAGAGC-3′ and reverse 5′-TGTGACCATTCACTCCAGGGGCT-3′.

### P and fH binding assays

We incubated PTECs with variable concentrations of P (0–10 μg/mL) or fH (0–400 μg/mL) for 3 h to examine the binding of P or fH to PTECs. To evaluate the competitive binding of P and fH, PTECs were also incubated with a mixture of variable concentrations of P (0–10 μg/mL) and fH (0–400 μg/mL) together for 3 h.

### Complement AP activation assay

Next, we examined AP activation, the enhancement effect of P and the dose-dependent effect of NHS on PTECs. PTECs were incubated with NHS (5% or 25%) for 3 h with or without preincubation with P at a fixed concentration (5 μg/mL) for 3 h. For further confirmation of the enhancement effect of P, we incubated PTECs with PDS (5% or 25%) for 3 h after preincubation with P (5 μg/mL).

### IF studies for complement components on PTECs

The IF studies were executed as described previously [20]. After incubation, PTECs on the cover glass of the 12-well culture plates were washed three times with 100 μL of phosphate-buffered saline (PBS) at a pH of 7.4. PTECs were then fixed with methanol for 5 min followed by washing with PBS three times. Subsequently, the cells were blocked with a blocking solution [1% bovine serum albumin (BSA) in PBS] at room temperature for 30 min. Indirect IF was used to determine P, fH and MAC. After removal of the blocking BSA, PTECs were stained with primary antibodies, i.e. mouse monoclonal anti-human P antibody (Ab) at a 1:100 dilution, sheep polyclonal anti-human fH Ab at a 1:50 dilution and rabbit polyclonal anti-human C5b-9 (MAC) Ab at a 1:300 dilution (Abcam, Cambridge, UK) for 1 h, respectively. After washing with PBS three times, PTECs were stained with secondary antibodies: Alexa Fluor 555-conjugated goat anti-mouse Ab at a 1:100 dilution (Invitrogen, Carlsbad, CA, USA), fluorescein isothiocyanate (FITC)-conjugated rabbit anti-sheep Ab at a 1:100 dilution (Abcam) and Alexa Fluor 488-conjugated donkey anti-rabbit Ab at a 1:250 dilution (Invitorogen) for 1 h, respectively. Direct IF was used to determine C3 deposition. After removal of the blocking BSA, PTECs were stained with FITC-conjugated rabbit anti-human C3c Ab at a 1:100 dilution (Dako, Glostrup, Denmark) for 1 h. The nuclei were stained with DAPI (4′,6-diamidine-2-phenylindole) at a 1:5000 dilution (SIGMA) for 5 min. The slides were washed with PBS several times and mounted. The deposition of complement components (P, fH, C3 and MAC) on PTECs was observed by IF using a confocal microscope (Olympus Viewer 1000, Olympus, Tokyo, Japan), and quantification of the depositions was evaluated by FV 10–ASW 2.1 imaging software (Olympus).

### Measurement of cell viability

We used the cell viability assay to assess the influence of complement activation and the enhancement effect of P on PTECs. PTECs were incubated with NHS (5% or 25%) or a serum-free medium (control) for 3 h with or without preincubation with P (5 μg/mL) for 3 h. Cell viability was assessed using the Cell Proliferation Kit I (MTT; Roche Applied Science, Penzberg, Germany) according to the manufacturer’s instructions. Briefly, PTECs placed in 96-well culture plates with 100 μL of culture medium (DMEM) were incubated with 10 μL of the MTT [3-(4,5-dimethylthiazol-2-2,5-yl)-2,5-diphenyltetrazolium bromide] solution in a CO_2_ incubator (5% CO_2_) at 37°C for 4 h. After this incubation, 100 μL of the solubilisation solution was added to each well and incubated in the CO_2_ incubator (5% CO_2_) at 37°C overnight. The spectrophotometrical absorbance of the samples was measured using a microplate reader (SpectraMax 340, Molecular Devices, Sunnyvale, CA, USA).

### Statistical analysis

The data are expressed as means ± standard deviations or standard errors. Comparisons among groups were performed using the Mann–Whitney U test or one-way ANOVA, and correlations among groups were evaluated by Spearman’s analysis using Graphpad Prism 5 software (Graphpad Software, La Jolla, CA, USA). Results of the multivariate analysis were assessed through multiple regression analysis using JMP software version 7 (SAS Institute Inc., Cary, NC, USA). A *p-*value of < 0.05 was considered statistically significant.

## Results

### *In vivo* studies

**Figure 1:**
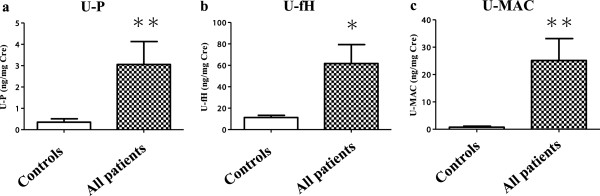
**Urinary (U)-properdin (P), factor H (fH) and membrane attack complex (MAC) levels U-P (a), fH (b) and MAC (c) levels were significantly higher in patients with renal diseases (n = 63) than in healthy controls (n = 48) as evaluated by ELISA.** **p < 0.0001 versus controls, *p < 0.05 versus controls.

In patients with renal diseases, the eGFR (mL/min/1.73 m^2^) and U-protein (g/g Cre) levels were 81.81 ± 29.54 and 2.13 ± 2.60, respectively. U-P, fH and MAC levels were significantly higher in patients with renal disease than in healthy controls, particularly U-P and MAC (*p* < 0.0001) levels (Figure 1). U-P, fH and MAC levels were strongly correlated with U-protein levels (*p* < 0.0001; Table 1). In addition, there were significant correlations of U-P, fH and MAC levels with the tubular damage markers U-B2MG (versus P, *p* < 0.0001; versus fH, *p* = 0.0013; versus MAC, *p* < 0.0005) and NAG (versus fH, *p* = 0.0158; versus MAC, *p* = 0.0003; Table 1). Moreover, U-protein levels correlated with U-B2MG (*p* = 0.001) and NAG (*p* < 0.0001) levels. Furthermore, multivariate analysis revealed the relationship between the U-complement components and tubular damage markers. U-B2MG was correlated with U-P and NAG was correlated with U-protein and U-P. U-P was the only item that was correlated with both B2MG and NAG (Table 2). Although patients with high U-protein levels, i.e. those with DMN, amyloidosis, MCNS, FSGS and MPGN, tended to have high U-P, fH and MAC levels, there was no significant correlation with the pathological diagnosis.

**Table 1:** Correlations of U-P, fH and MAC with clinical markers in patients with renal diseases

	**P**		**fH**		**MAC**	
	**r**	***p***	**r**	***p***	**r**	***p***
U-protein	0.756	<0.0001^***^	0.525	<0.0001^***^	0.577	<0.0001^***^
U-B2MG	0.675	<0.0001^***^	0.401	0.0013^**^	0.431	<0.0005^**^
U-NAG	0.247	0.0502	0.303	0.0158^*^	0.443	0.0003^**^
eGFR	−0.392	0.0015^**^	−0.319	0.0106^*^	−0.19	0.1348

*Abbreviations*: U, urinary; P, properdin; fH, factor H; MAC, membrane attack complex; U-B2MG, U-beta-2-microglobulin; U-NAG, U-N-acetyl-β-D-glucosaminidase; eGFR, estimated glomerular filtration rate.

**Table 2:** Association of U-P, fH, MAC and U-protein as independent variables and U-B2MG and NAG as response variables in patients with renal diseases

**Response variables**	**Independent variables**	**OR**	**95% CI**	***p***
U-B2MG	U-P	5090.86	2305.25 to 7878.46	0.0006^**^
	U-fH	253.11	−2353.81 to 1847.58	0.8101
	U-MAC	1200.49	−1350 to 3751.50	0.3499
	U-protein	912	−12353.81 to 1847.58	0.3986
U-NAG	U-P	−2.32	−4.22 to −0.43	0.0168^*^
	U-fH	0.03	−1.47 to 1.55	0.9583
	U-MAC	1.7	−0.15 to 3.55	0.0712
	U-protein	3.15	1.58 to 4.71	0.0002^**^

*Abbreviations*: P, properdin; fH, factor H; MAC, membrane attack complex; U-B2MG, U-beta-2-microglobulin; U-NAG, U-N-acetyl-β-D-glucosaminidase; OR, odds ratio; CI, confidence interval.

### *In vitro* studies

#### mRNA expressions of complement components belonging to the AP of PTECs

**Figure 2:**
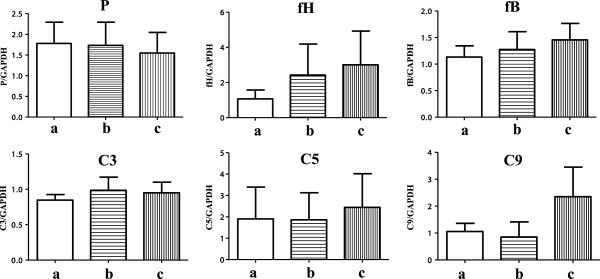
**mRNA expressions of complement components belonging to the alternative pathway (AP) by proximal tubular epithelial cells (PTECs).** PTECs were incubated with different concentrations of normal human serum (NHS; 5% or 25%) for 3 h. There were no significant changes in the mRNA expression of AP components (P, fH, fB, C3, C5 and C9) after the addition of up to 25% NHS. **(a)** Serum-free medium, **(b)** 5% NHS and **(c)** 25% NHS as evaluated by real-time polymerase chain reaction. Data are expressed as relative values compared with those for control cells after the addition of a serum-free medium for 0 h. The results are expressed as means ± standard errors of values from four independent experiments.

There was no significant upregulation of mRNA expression of the AP components, P, fH, fB, C3, C5 or C9 in PTECs after the addition of up to 25% NHS for 3 h (Figure 2). These results suggested that there was no significant overproduction of the complement AP components by PTECs after the addition of up to 25% NHS.

### Binding of P and fH on PTECs

**Figure 3:**
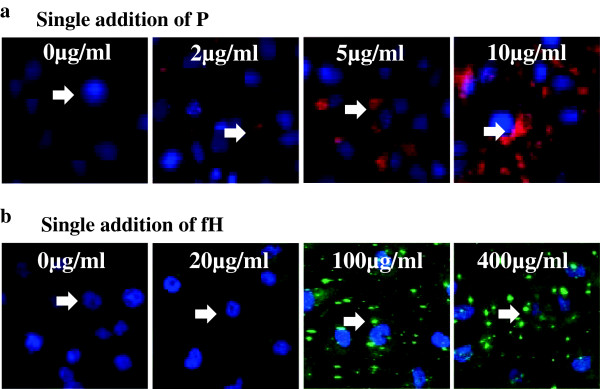
**Binding of properdin (P) and factor H (fH) on proximal tubular epithelial cells (PTECs).** PTECs were incubated with variable concentrations of P (0–10 μg/mL) or fH (0–400 μg/mL) for 3 h. Dose-dependent depositions of P **(a)** or fH **(b)** were observed by immunofluorescence (IF). Depositions of P and fH were observed at a concentration (P, ≥2 μg/mL; fH, ≥20 μg/mL) lower than the physiological serum concentration (P, 4–6 μg/mL; fH, 300–500 μg/mL). Original magnification: ×100. Arrow: depositions of P or fH.

Dose-dependent depositions of P and fH were observed after the addition of P and fH. Depositions of P and fH were observed at a concentration (P, ≥2 μg/mL; fH, ≥20 μg/mL) lower than the physiological serum concentration (P, 4–6 μg/mL; fH, 300–500 μg/mL; Figure 3) [21,22].

### Competitive binding of P and fH on PTECs

**Figure 4:**
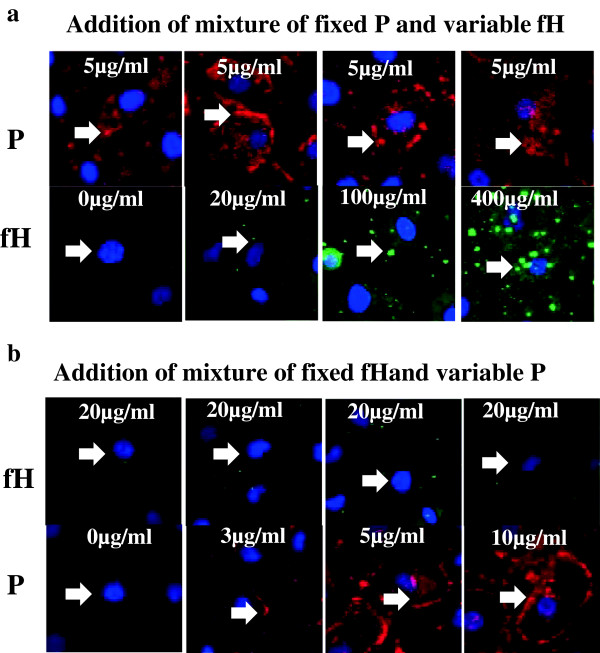
**Competitive binding of properdin (P) and factor H (fH) on proximal tubular epithelial cells (PTECs).** PTECs were incubated with variable concentrations of a mixture of P and fH for 3 h. **(a)** In the mixture of P and fH (P concentration, 5 μg/mL; fH concentration, 0–400 μg/mL), the depositions of fH were increased in a dose-dependent manner, while those of P remained unchanged, as observed by IF. **(b)** In the mixture of P and fH (fH concentration, 20 μg/mL; P, 0–10 μg/mL), the depositions of P were increased in a dose-dependent manner, while those of fH remained unchanged. Original magnification: ×100. Arrow: depositions of P or fH.

When the concentrations of P were fixed (5 μg/mL) and those of fH were variable (0–400 μg/mL), the depositions of fH were dose-dependent, while those of P were not inhibited by the variable concentrations of fH (Figure 4a). On the other hand, when the concentrations of fH were fixed (20 μg/mL) and those of P were variable (0–10 μg/mL), the depositions of P were dose-dependent, while those of fH were not inhibited by the variable concentrations of P (Figure 4b).

### Complement AP activation on PTECs in a serum-dependent manner

**Figure 5:**
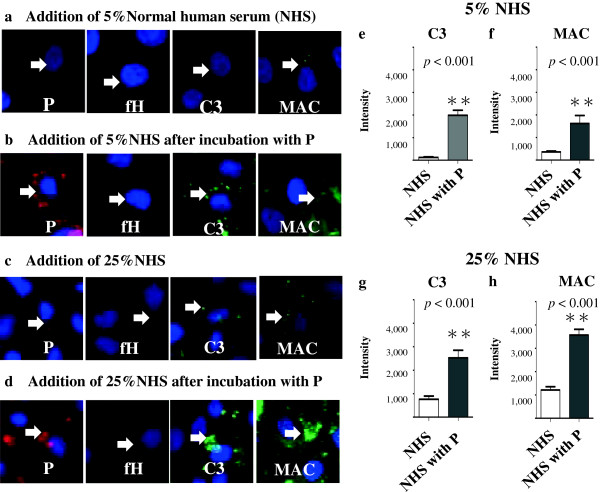
**Complement alternative pathway (AP) activation on proximal tubular epithelial cells (PTECs).** PTECs were incubated with different concentrations of normal human serum (NHS; 5% or 25%) for 3 h. Depositions of properdin (P), factor H (fH), C3 and membrane attack complex (MAC) with 25% NHS **(c)** were stronger than those with 5% NHS **(a)**, as observed by immunofluorescence (IF). Preincubation with P (5 μg/mL) for 3 h before the addition of both 5% **(b)** and 25% **(d)** NHS increased the depositions of P, C3 and MAC compared with those with 5% **(a)** and 25% **(c)** intact NHS. Quantitative analysis was performed, and preincubation with P before the addition of both 5% and 25% NHS significantly increased depositions of C3 **(e, g)** and MAC **(f, h)** (**p < 0.001) compared with the addition of 5% and 25% intact NHS only. Results are expressed as means ± standard errors of values from five representative lesions. Original magnification: ×100 Arrow: depositions of P, fH, C3 or MAC.

Depositions of P, fH, C3 and MAC were observed after the addition of both 5% and 25% NHS. Depositions of P, fH, C3 and MAC were stronger with 25% NHS than with 5% NHS. Preincubation with the fixed concentration of P (5 μg/mL) before the addition of both 5% and 25% NHS increased the depositions of P, C3 and MAC compared with the addition of 5% and 25% intact NHS only (Figures 5a,b,c,d); preincubation also increased the depositions of C3 and MAC in a serum-dependent manner from 5% to 25% NHS. Quantitative analysis was performed, and preincubation with P before the addition of both 5% and 25% NHS significantly increased (*p* < 0.001) the depositions of C3 and MAC compared with the addition of 5% and 25% intact NHS only (Figures 5e,f,g,h)

### Enhancement effects of P for complement AP activation on PTECs

**Figure 6:**
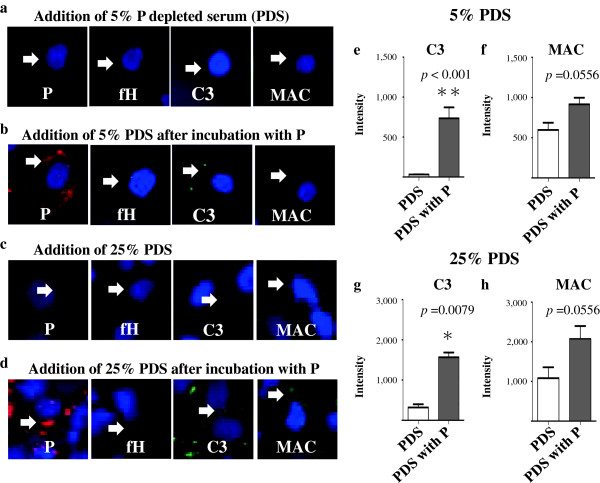
**Enhancement effect of properdin (P) in complement alternative pathway (AP) activation on proximal tubular epithelial cells (PTECs), PTECs were incubated with P-depleted serum (PDS; 5% or 25%) for 3 h.** Preincubation with P (5 μg/mL) for 3 h before the addition of PDS increased the depositions of P, C3 and MAC **(b, d)** compared with the addition of intact PDS only **(a, c)**. Depositions of P were slightly detected with the addition of 25% PDS, but not 5% PDS. Quantitative analysis was performed, and preincubation with P before the addition of PDS significantly increased the depositions of C3 **(e, g)** (5% PDS, **p < 0.001; 25% PDS, *p = 0.0079) and relatively increased the depositions of MAC (*p* = 0.0556) **(f, h)** compared with the addition of intact PDS only. Results are expressed as means ± standard errors of values from five representative lesions. Original magnification: ×100. Arrow: depositions of P, fH, C3 or MAC.

Preincubation with P before the addition of 5% and 25% PDS increased the depositions of P, C3 and MAC compared with the addition of 5% and 25% intact PDS only (Figures 6a,b,c,d). Depositions of P were slightly detected with the addition of 25% PDS, but not 5% PDS. Quantitative analysis was performed, and preincubation with P before the addition of 5% and 25% PDS significantly increased the depositions of C3 (5%, *p* < 0.001; 25%, *p* = 0.0079) and relatively increased the depositions of MAC (*p* = 0.0556) compared with the addition of 5% and 25% intact PDS only (Figures 6e,f,g,h).

### Morphological changes and viability of PTECs

**Figure 7:**
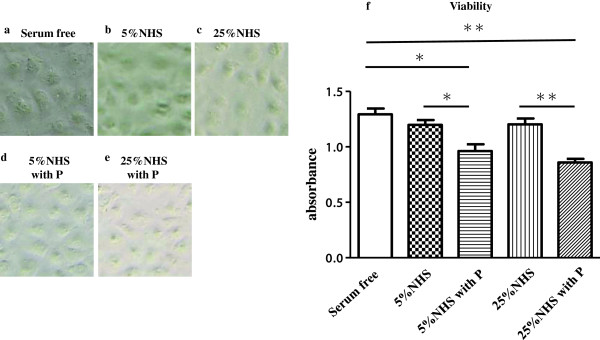
**Morphological changes and viability of proximal tubular epithelial cells (PTECs).** PTECs were incubated with normal human serum (NHS; 5% or 25%) or serum-free medium (controls) for 3 h with or without preincubation with P (5 μg/mL) for 3 h. There were no significant morphological changes in PTECs after the addition of up to 25% NHS. Preincubation with P before the addition of both 5% and 25% NHS did not cause morphological changes in PTECs. **(a)** Serum-free medium (control), **(b)** 5% NHS, **(c)** 25% NHS, **(d)** 5% NHS with preincubation with P and **(e)** 25% NHS with preincubation with P, as observed by a microscope. Pictures are representative of four-cell culture plates. Original magnification: ×50. There were no significant changes in the viability of PTECs after the addition of 5% and 25% NHS **(f)**. Preincubation with P before the addition of both 5% and 25% NHS significantly suppressed the viability of PTECs compared with controls and 5% and 25% intact NHS **(f)** (**p < 0.01, *p < 0.05). Results are expressed as means ± standard errors of values from eight independent experiments.

There were no significant morphological changes and viability changes in PTECs after the addition of up to 25% NHS (Figure 7a,b,c,f). Preincubation with P before the addition of both 5% and 25% NHS significantly suppressed the viability of PTECs compared with controls and 5 and 25% intact NHS, without morphological changes (Figure 7a,d,e,f).

## Discussion

This study aimed to clarify the role of AP regulation in renal tubular damage through both *in vivo* and *in vitro* examinations. First, we demonstrated that U-P, fH and MAC levels were significantly higher in patients with various renal diseases than in healthy controls and were correlated with U-protein levels and tubular damage markers. In addition, U-protein levels were correlated with the tubular damage markers, and the U-P, fH and MAC levels were independent of the histological diagnosis, so that the filtered plasma protein through glomerular barrier impacted as an important causative factor in renal tubular damage [2,9,23,24]. Even in a patient with MCNS who had decreased renal function (eGFR: 60.4 mL/min), there was a tendency of high levels of U-tubular damage markers, B2MG 9014.28 ng/mg Cre (all patients: 1176 ± 3113) and NAG 5.41 U/mg Cre (all patients: 1.57 ± 2.08), U-protein 10.38 g/g Cre and U-complement components, U-P 44.10 ng/mg Cre, and U-fH 254.22 ng/mg Cre and U-MAC 62.59 ng/mg Cre. Although there were no high levels of tubular damage markers and U-complement components in patients without decreased eGFR in MCNS, these results suggested that relationships among high levels of U-protein, U-complement components and U-tubular damage markers existed, even in MCNS patients. Therefore, as U-complement components and tubular damage markers increase in patients with MCNS and advanced renal dysfunction, the levels of U-complement components and tubular damage markers may correlate with the levels of U-protein. Furthermore, multivariable analysis revealed that only U-P was correlated with both tubular damage markers, B2MG and NAG. In the context of renal disease, this study represents the first analysis, as per our knowledge, to clarify the role of AP regulation by measuring the combination of U-P, fH and MAC in the same samples from patients with various renal diseases.

The question arises whether these U-complement components play a crucial role in renal tubular damage. We examined complement AP activation on PTECs and could delineate a delicate balance between P and fH. From the single addition of P or fH, we could confirm dose-dependent depositions of P and fH on PTECs [14,19,23]. Zaferani et al. [14] suggested that P could bind dose-dependently to heparan sulfate proteoglycan (HSPG), which was thought to be one of the binding sites on PTECs. They also suggested [23] that fH could bind dose-dependently to HSPG and that P and fH could bind to different epitopes of HSPG on PTECs. Our results support these previous reports and indicated that P and fH could bind dose-dependently to PTECs. Although the depositions of fH were observed in a concentration (≥20 μg/mL) lower than the physiological serum concentration of fH (300–500 μg/mL), the bindings of P and fH are thought to be non-competitive in this experiment. Unfortunately, the individual binding site on PTECs could not be identified in this experiment, but it does suggest that the binding of fH does not have enough power to prevent the binding of P on PTECs.

We then recaptured the activation of complement AP on PTECs. NHS was used as a source of complements and a mimic of filtered plasma protein through the glomerular barrier. The depositions of P, fH, C3 and MAC on PTECs were observed to be dose-dependent of NHS [9,25]. Next, to examine the enhancement effect of P in AP activation on PTECs, we preincubated PTECs with P before the addition of NHS. Preincubation with the fixed concentration of P before the addition of NHS increased not only the depositions of P but also those of C3 and MAC compared with controls; preincubation also increased the depositions of C3 and MAC in a serum-dependent manner from 5% to 25% NHS. Gaarkeuken et al. [9] also reported that the filtered P may bind to PTECs and act as a focal point for AP activation. In that report, P could be detected along the brush border of the proximal tubules in the patients with MN, but it was not detected in the tubules of healthy kidney tissues (pretransplant renal biopsies of living kidney donors). Additional P was shown to increase the depositions of C3 and MAC on PTECs in a P dose-dependent manner before incubation with 5% NHS. These findings could add new insight to the previous reports by suggesting that PTEC could initiate complement activation in a serum-dependent manner, with pre-exposure to a physiological concentration of P.

We previously focused on the importance of PDP activation in the pathogenesis of LN and reported that PDP activation in the glomeruli may lead to the early onset and progression of LN and reflect disease activity [26]. In this study, we also confirmed that preincubation with P before the addition of PDS increased not only the deposition of P but also the deposition of C3, while it relatively increased the deposition of MAC; it was also suggested that P may act as an enhancer of complement AP activation referred to as PDP activation [17]. A similar result was confirmed using flow cytometry in a previous report [9], where additional P restored complement activation of PDS on PTECs. We unexpectedly detected slight P staining on PTECs incubated with 25% PDS, but not 5% PDS. We considered the possibility that PDS was not completely depleted of P in the serum and that even a very small amount of P could bind to PTECs and activate AP and PDP, leading to the deposition of MAC. We could confirm that 5.77 ng/mL of P was present in the 25% PDS by ELISA (physiological serum concentration of P: 4–6 μg/mL).

In our study, although the activation of AP was seemingly ineffective in morphology, a functional effect was observed. After the addition of NHS to PTECs, cell viability was not significantly suppressed; however, preincubation with P before the addition of NHS significantly suppressed viability without causing morphological changes. Although diverse factors affected cell viability [1], the changes in this experiment were thought to reflect one possible result of complement AP activation enhanced by P. Therefore, we demonstrated the relationship between AP activation enhanced by P and tubular damage in experimental situations.

While there are numerous reports, including this study, that support the role of P in AP complement-mediated renal tubular damage, P-targeting therapy has also been evaluated [11,27,28]. P blockade may be a logical therapeutic target, and the accumulation of further research is required to decrease renal tubular damage.

## Conclusions

In the pathogenesis of renal tubular damage, P can bind directly to PTECs and may play a significant role in renal tubular damage by accelerating complement AP activation and surpassing fH regulation.

## Competing interests

The authors declare that they have no competing interests.

## Authors’ contributions

SN collected samples and prepared materials, conducted the study, analysed the data and wrote the manuscript. IO (principal investigator) offered advice on the study and reviewed the manuscript. HS, NS and YS helped in conducting the study. AH, DH and MS helped in sample collection. HI reviewed the manuscript. SH participated in the design of the study. YT (primary principal investigator) offered advice on the study. All authors have read and approved the final manuscript.

## Pre-publication history

The pre-publication history for this paper can be accessed here:

http://www.biomedcentral.com/1471-2369/15/82/prepub
